# Spontaneous Lens Absorption Initially Misdiagnosed as Crystalline Lens Luxation

**DOI:** 10.4274/tjo.65725

**Published:** 2018-12-27

**Authors:** Şaban Gönül, Ayşe Bozkurt Oflaz, Berker Bakbak, Kamil Yavuzer, Banu Bozkurt

**Affiliations:** 1 Selçuk University Faculty of Medicine, Department of Ophtalmology, Konya, Turkey; 2 Adana City Training and Research Hospital, Ophtalmology Clinic, Adana, Turkey; 3 Van Regional Training and Research Hospital, Ophtalmology Clinic, Van, Turkey

**Keywords:** Fuchs’ uveitis syndrome, hypermature cataract, spontaneous absorption

## Abstract

Spontaneous lens absorption (SLA) is a rare complication of hypermature cataract. However, this condition has been reported in several cases of hypermature cataracts that were caused by trauma, senility, uveitic disorders such as Fuchs’ uveitis syndrome (FUS), and infectious disorders including leptospirosis and rubella. We report a case of spontaneous absorption of a hypermature cataract secondary to FUS. To our knowledge, this is the first report of SLA that was followed by dislocation of the capsular remnants into the vitreous and resulted in a misdiagnosis as crystalline lens luxation.

## Introduction

Spontaneous lens absorption (SLA) is a rare pathology, especially in recent years. It can occur in hypermature and traumatic cataract or in the late stages of some uveitic and infectious diseases.^1,2,3,4,5,6^ Recent advances in cataract surgery and the increasing use of surgery have reduced the incidence of hypermature cataracts, thus reducing the prevalence of SLA. In this case report, we present the clinical features of a patient who developed SLA secondary to Fuchs’ uveitis syndrome (FUS), a rarely seen entity in our clinical practice.

## Case Report

A 63-year-old female patient presented with reduced vision in her right eye. She reported experiencing sudden-onset pain, loss of vision, and redness in her right eye 7 years earlier, but did not seek medical treatment at that time. She had no history of ocular trauma or surgery. Best corrected visual acuity (BCVA) in her right eye was light perception and intraocular pressure was 18 mmHg. Anterior segment examination revealed hypermature cataract. The iris stroma showed diffuse atrophy and appeared hypochromic. Ultrasonography demonstrated retinal attachment. Cataract surgery was recommended, but the patient refused.

**Figure 1:**
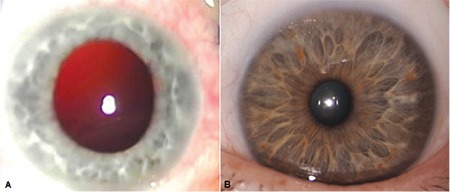
The patient’s right eye (A) appears aphakic with iris hypochromia; the left eye (B) shows iris hyperpigmentation

**Figure 2:**
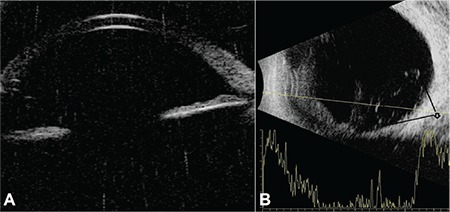
Ultrasound biomicroscopy image of the right eye (A) shows no findings of crystalline lens material posterior of the iris. B-scan ultrasonography (B) shows a hyperechoic area posterior of the iris in the lower quadrant that suggests luxation of the crystalline lens

**Figure 3:**
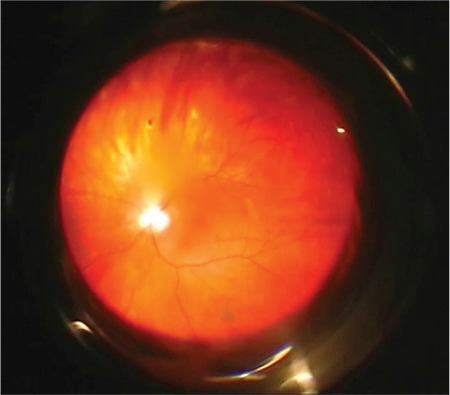
Retinal image obtained during pars plana vitrectomy does not show any crystalline lens material

**Figure 4:**
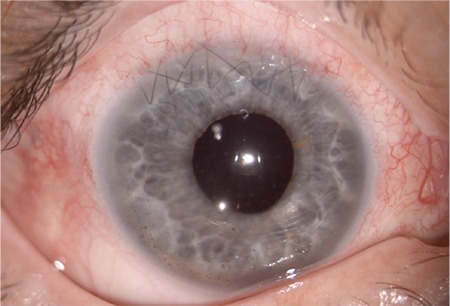
Corneal-scleral sutures, centralized intraocular lens, and clear cornea are seen in postoperative day 1 examination

At 1-year follow-up examination, the patient stated that her vision had improved. BCVA was 20/25 in the right eye (with +12 D correction) and 20/20 in the left eye. Although her right eye appeared aphakic on anterior segment examination, no surgical scar or signs of trauma were detected. The cornea was clear and the conjunctiva appeared normal. Despite the hyperchromic appearance and stroma atrophy of the iris, there were no findings suggestive of inflammation (keratic precipitates in the corneal endothelium, posterior synechia, or anterior chamber inflammatory cells). The left eye appeared normal (Figure 1). Intraocular pressure was 18 mmHg in the right eye and 16 mmHg in the left eye. The areas that could be visualized in fundus examination were normal. A peripheral retinal scan was done to see the crystalline lens. An ideal evaluation could not be performed because the patient had sunken eyes and incomplete pupil dilation. However, no crystalline lens material was observed in the visualized areas. The absence of crystalline lens material in peripheral retinal examination raised the suspicion of crystalline lens subluxation behind the iris. Ultrasound biomicroscopy (UBM) was performed, but UBM images did not show any lens material behind the iris (Figure 2A). B-scan ultrasound revealed a hyperechoic appearance in the inferior peripheral retina suggesting luxation (Figure 2B). Based on these findings, the patient was scheduled for 23-gauge pars plana vitrectomy. Despite intraoperative investigation using 360° scleral depression, there were no signs of the crystalline lens (Figure 3). During the procedure, atrophic holes formed in the peripheral retina. Prophylactic 360° laser was applied to the peripheral retina at the end of the procedure. An intraocular lens was implanted in the posterior chamber by scleral fixation (Figure 4). Postoperatively, the possible etiologies of SLA were investigated. Toxoplasma and leptospirosis tests were negative, while cytomegalovirus, rubella, and herpes simplex virus IgG antibodies were positive. Sedimentation rate, complete blood count, urinalysis, and biochemical tests were within normal limits. The patient was followed for 1 year with no complications.

## Discussion

SLA is a rarely encountered pathology in clinical practice. Hypermature cataracts secondary to trauma, advanced age, and certain uveitic and infectious diseases may cause SLA.^1,2,3,4,5,6^ The mechanism underlying SLA has not been fully elucidated. In leptospirosis and rubella infection, it is thought to be a response to the agent itself or to antibodies, though the mechanism has not been established.^4^ Absorption is unlikely in the presence of an undamaged lens capsule, but may occur following damage to the lens capsule in late hypermature cataract.^1^ Similarly, our patient had hypermature cataract. Structural and chemical events associated with FUS also likely facilitated this process.

Before making a clinical diagnosis of SLA, subluxation or lens migration into the vitreous must be ruled out. In addition, the absence of scarring associated with ocular trauma or previous ocular surgery should be confirmed during examination.^1^ Our patient had a clear cornea and exhibited no signs of trauma. There were no findings in UBM suggesting lens subluxation. B-scan ultrasound revealed a hyperechoic image thought to be the crystalline lens lumen. Therefore, we suspected lens migration into the vitreous.

In a case of SLA caused by hypermature cataract following FUS, Uemura et al.^3^ reported that the presence of capsular remnants in the anterior chamber facilitated the diagnosis. Kobat et al.^7^ also presented a case of SLA after hypermature cataract in which capsule pieces found in the anterior chamber provided diagnostic clues. Similarly, Kim et al.^8^ discussed capsule remnants in the anterior chamber in their patients with SLA. In our case, however, no such remnants were observed in the anterior chamber. This delayed an accurate diagnosis.

While the definitive etiopathogenesis of SLA remains unclear, a possible association with infections such as leptospirosis and rubella has been emphasized in several publications.^4,5,6^ It has also been reported that FUS may be associated with rubella infection as well as be involved the etiology of SLA.^9,10^ Our patient tested positive for rubella IgG. No intraocular sampling of the aqueous humor was done to assess for antibodies to the rubella genome or virus to determine the association between the past rubella infection and both FUS and SLA. For this reason, we were unable to prove a direct causal relationship between FUS, SLA, and her prior rubella infection.

In conclusion, the migration of capsular remnants into the vitreous after SLA is a rare complication of hypermature cataract. This can lead to a misdiagnosis of crystalline lens luxation, especially when conditions for peripheral retinal examination are not ideal. Therefore, SLA should be kept in mind during the clinical evaluation of such patients.
